# Neddylation inhibitor MLN4924 sensitizes head and neck squamous carcinoma cells to (S)-10-hydroxycamptothecin

**DOI:** 10.1186/s40001-023-01289-y

**Published:** 2023-09-09

**Authors:** Shanshan Gu, Chen Lin, Yanguo Li, Zhengyu Wei, Bing cao, Zhisen Shen, Hongxia Deng

**Affiliations:** 1 Department of Otorhinolaryngology Head and Neck Surgery, Lihuili Hospital affiliated to Ningbo University, Ningbo, 315040 Zhejiang China; 2 School of Medicine, Ningbo University, Ningbo, China; 3 Institute of Drug Discovery Technology, Ningbo University, Ningbo, China

**Keywords:** HNSCC, 10-HCPT, TOP1, MLN4924

## Abstract

Head and neck squamous carcinoma (HNSCC) is the seventh most common cancer worldwide. Targeted therapeutic drugs for HNSCC are still being explored. Among them, (S)-10-hydroxycamptothecin (10-HCPT), a specific inhibitor of TOP1, functions by DNA double-strand breaks that can inhibit DNA replication and trigger apoptotic cell death subsequently. Previous studies have reported that MLN4924 exerts potent anti-tumor effects by inhibiting cullin–RING ligases and causing substrate accumulation in a variety of cancers. Here, we show that MLN4924 effectively causes dose-dependent accumulation of topoisomerase I (TOP1) and blocks TOP1 ubiquitination. Importantly, neddylation inhibition with MLN4924 acts synergistically with 10-HCPT to suppress cell growth, migration and apoptosis in HNSCC cells. Mechanistically, transcriptome sequencing shows that the cytotoxic effects of the combination of MLN4924 and 10-HCPT may involve activation of the NFKB1 pathway. Taken together, our results suggest that combined treatment with MLN4924 and 10-HCPT may be an effective strategy in HNSCC.

**Supplementary Information:**

The online version contains supplementary material available at 10.1186/s40001-023-01289-y.

## Introduction

Head and neck cancer is the seventh most common malignancy worldwide, with an annual incidence of more than 890,000 cases. More than 90% of head and neck tumors are squamous cell carcinoma (HNSCC) [1, 2]. The global incidence, which is rapidly increasing year by year, is associated with exposure to carcinogens (alcohol and/or tobacco) and high-risk HPV infection [3]. Although treatment with conventional chemotherapy has shown clinical benefits in HNSCC patients, the 5-year survival rate remains unsatisfactory owing to treatment-induced toxicities and drug resistance [4]. Thus, there is an urgent and critical need to develop innovative targeted therapeutic agents that sensitize HNSCC to chemotherapy and improve the therapeutic efficacy.

(S)-10-hydroxycamptothecin (10-HCPT), originally detached from a Chinese tree, *Camptotheca cuminata*, is an indole alkaloid derived from camptothecin [5]. 10-HCPT, a specific inhibitor of TOP1, binds to DNA and DNA enzyme TOP1 and forms a ternary complex, leading to DNA double-strand breaks that inhibit DNA replication and trigger apoptotic cell death [6, 7]. 10-HCPT has been reported to show potent anti-tumor activity in HNSCC [8]; however, acquired clinical resistance and digestive tract reactions are common. Previous studies have indicated that Cul3 promotes proteasomal degradation of covalent TOP1–DNA complexes, resulting in camptothecin resistance [9, 10]. Thus, we investigated whether inhibition of TOP1 degradation using NEDD8-activating enzyme inhibitor MLN4924 could result in better anti-tumor effects of 10-HCPT in HNSCC treatment.

Protein neddylation is an important post-translational modification that tags neural precursor-cell-expressed developmentally downregulated protein 8 (NEDD8) onto specific substrates to modulate protein function. This reaction is a sequential enzymatic process catalyzed by NEDD8-activating enzyme E1 (NAE), NEDD8-conjugating enzyme E2 and NEDD8-E3 ligases [11, 12]. NEDD8 is a ubiquitin-like molecule that modulates the activities of cullin–RING ligases (CRLs). Hyperactivation of neddylation pathways and dysfunction of CRLs E3 ubiquitin ligases have been implicated in many human cancers, indicating the potential of these pathways and ligases as anti-cancer therapeutic targets. MLN4924, an adenosylamino sulfonate derivative, is a potent and selective inhibitor of NAE. It specifically binds to NAE and forms a MLN4924–NEDD8 adduct, resulting in inhibition of CRLs and accumulation of substrates [13, 14]. Several studies have shown that MLN4924 induces apoptosis, senescence, and autophagy, suppressing the growth of tumor cell both in vitro and in vivo [11, 15, 16]. Having shown promising anti-cancer activity in preclinical models, MLN4924 has been tested in phase I/II clinical trials for the treatment of patients with leukemia, lymphoma, melanoma, and several solid tumor types [11]. MLN4924 proved able to efficiently sensitize tumors to chemoradiation therapies, and a combination of MLN4924 and azacitidine for the treatment of adult acute myeloid leukemia patients is under investigation in phase 1b/2 trials. Therefore, we hypothesized that a combination of 10-HCPT and MLN4924 could represent an attractive anti-cancer strategy for the treatment of HNSCC. Our results demonstrate that MLN4924 effectively enhances the suppression of cell growth, migration, and apoptosis by 10-HCPT in HNSCC cells. These findings provide insights into the critical role of MLN4924 and establish a novel targeted therapeutic strategy for HNSCC.

## Materials and methods

### Cell lines and chemicals

Laryngeal carcinoma cell line AMC-HN-8 and tongue cancer cell line CAL-27 were obtained from the BeNa Culture Collection (Xinyang City, Henan Province, China), and HEK293 cells were obtained from the American Type Culture Collection (Manassas, VA, USA). Cells were cultured in Dulbecco’s modified Eagle medium (DMEM) containing 10% fetal bovine serum (FBS) and 1% penicillin–streptomycin at 37 °C in a 5% CO_2_ incubator. (S)-10-hydroxycamptothecin was purchased from Selleck (S2423) and MLN4924 from Apexbio (B1036); Both were dissolved in dimethyl sulfoxide (DMSO) and stored at − 20 °C.

### Cell viability and clonogenic survival assays

AMC-HN-8 and Cal-27 cells were seeded in 96-well plates (3 × 10^3^ cells per well) in triplicate. Cells were treated with different concentrations of 10-HCPT alone or in conjunction with MLN4924. After incubation for 24 h, cell viability was assessed with an ATP-lite Luminescence Assay kit (PerkinElmer) according to the manufacturer’ s instructions.

For clonogenic survival assays, 350 cells were plated in 60-mm dishes in triplicate for 24 h and then treated with 10-HCPT, MLN4924, or a combination (10-HCPT + MLN4924) for 7–14 days. Colonies were fixed with Coomassie Brilliant Blue solution for 20 min and carefully washed with running water. Finally, colonies were photographed and manually counted (colonies containing more than 50 cells were included in the final count).

### Western blotting

Cells were harvested and lysed in lysis buffer (1% NP-40, 0.1% sodium dodecyl sulfate (SDS), 0.5% sodium deoxycholate, 50 mM Tris pH 7.5, 0.15 M NaCl, 50 mM NaF, 1 mM EDTA, 1 mM DTT, 1 mM Na_3_VO_4_) with proteasome and phosphatase inhibitors. Following a 30-min incubation on ice, the supernatant was collected by centrifugation at 13,600 rpm for 25 min at 4 °C. Protein samples were separated by SDS polyacrylamide gel electrophoresis, transferred to nitrocellulose membranes and incubated with the appropriate primary and secondary antibodies. Proteins were detected using SuperSignal™ West Pico PLUS Chemiluminescent Substrate (Thermo, 34,580). The following primary antibodies were used: TOP1 (ab3825, Abcam), cyclin B1 (12231#), caspase-3 (9665#), cleaved caspase-3 (9661#), PARP (9542#), cleaved-PARP (5625#), p-CHK1 (S345) (2348#), P21 (2947#) (Cell Signaling Technology), CUL 1 (sc-17775) and t-CHK1 (sc-8408) (Santa Cruz), actin (A5441#) and FLAG (F1804) (Sigma).

### Wound-healing assay

Cells were seeded in six-well plates, and scraped across the wells with a 10 µL sterile pipette tip. Detached cells were removed by washing with phosphate-buffered saline (PBS). Then, after treatment with MLN4924 (50 nM) and/or 10-HCPT (5 nM) for 24 h, cells were photographed at 0 h, 24 h and 48 h. The width of the wound was measured using Image J software.

### Transwell migration and invasion assays

Cells were starved for 24 h and then seeded into a 24-well plate, of which the upper transwell chamber contained serum-free DMEM and the bottom well contained DMEM with 10% FBS. For the invasion assay, the upper chamber was filled with Matrigel matrix. After a 24-h incubation, the cells were stained with 0.05% crystal violet (Aladdin, C110703) for 20 min. Then, the migrating or invasive cells on the bottom of the membrane were photographed and counted in five random microscopic fields.

### Flow cytometry

Cells were treated with the various concentrations of 10-HCPT, MLN4924, or a combination of both. Then, cells were washed with PBS, trypsinized, and subjected to staining with an Annexin V-FITC apoptosis detection kit (Beyotime, C1063) according to the manufacturer’s instructions. Flow cytometry was used to detect cell apoptosis. All experiments were performed in triplicate.

### Sample collection and RNA sequencing

Samples were collected in triplicate from four experimental groups: control (DMSO), MLN4924 (1 µM), 10-HCPT (0.5 µM), and MLN4924 (1 µM) and 10-HCPT (0.5 µM) in combination. Total RNA was extracted using TRIzol reagent (Invitrogen). RNA purity and integrity were evaluated and quantification was performed with an Agilent 2100 Bioanalyzer. Following library construction, transcriptome sequencing was performed on an illumina NovaSeq 6000 platform.

### Prognostic analysis

We performed a retrospective analysis of patient survival data using a publicly available dataset from The Cancer Genome Atlas (TCGA-HNSC) obtained through UCSC Xena. Our methodology involved subgroup stratification of patients into four categories based on median expression levels of TOP1 and NEDD8, followed by the development of an accurate prognostic model. Then, visual representations of survival effects were generated using Kaplan–Meier plots.

### Statistical analysis

Data are presented as mean ± SEM. We used Student's t-test in SPSS version 20.0 (IBM Corporation, Armonk) to determine statistical significance. Values of p < 0.05 were considered to indicate statistical significance.

## Results

### Expression levels and prognostic value of NEDD8 and TOP1 in HNSCC

**Fig. 1:**
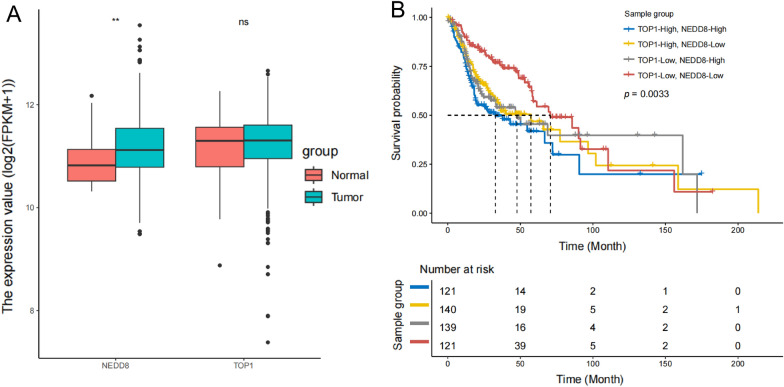
Expression levels and prognostic value of NEDD8 and TOP1 in HNSCC. **A** NEDD8 expression was significantly elevated in HNSCC tumors compared with normal tissues from TCGA. Mean ± SD. **B** Survival analysis for patients in high and low groups expression groups by median. **p < 0.01

Transcriptome analysis of data obtained from TCGA showed significant upregulation of NEDD8 in HNSCC compared with normal tissue. However, expression levels of TOP1 showed no significant difference between the two tissue types, indicating that traditional analysis based on differential genes may overlook the crucial role of TOP1 (Fig. 1A). We then created a prognostic model demonstrating that low levels of NEDD8 and TOP1 expression in tumor tissues are associated with a favorable prognosis (p < 0.01, Fig. 1B). These findings suggest that TOP1 and NEDD8 could have a synergistic effect on tumor progression and patient prognosis. Thus, inhibiting their expression may improve patient outcomes.

### MLN4924 causes a dose-dependent accumulation of TOP1 and blocks TOP1 ubiquitination.

**Fig. 2:**
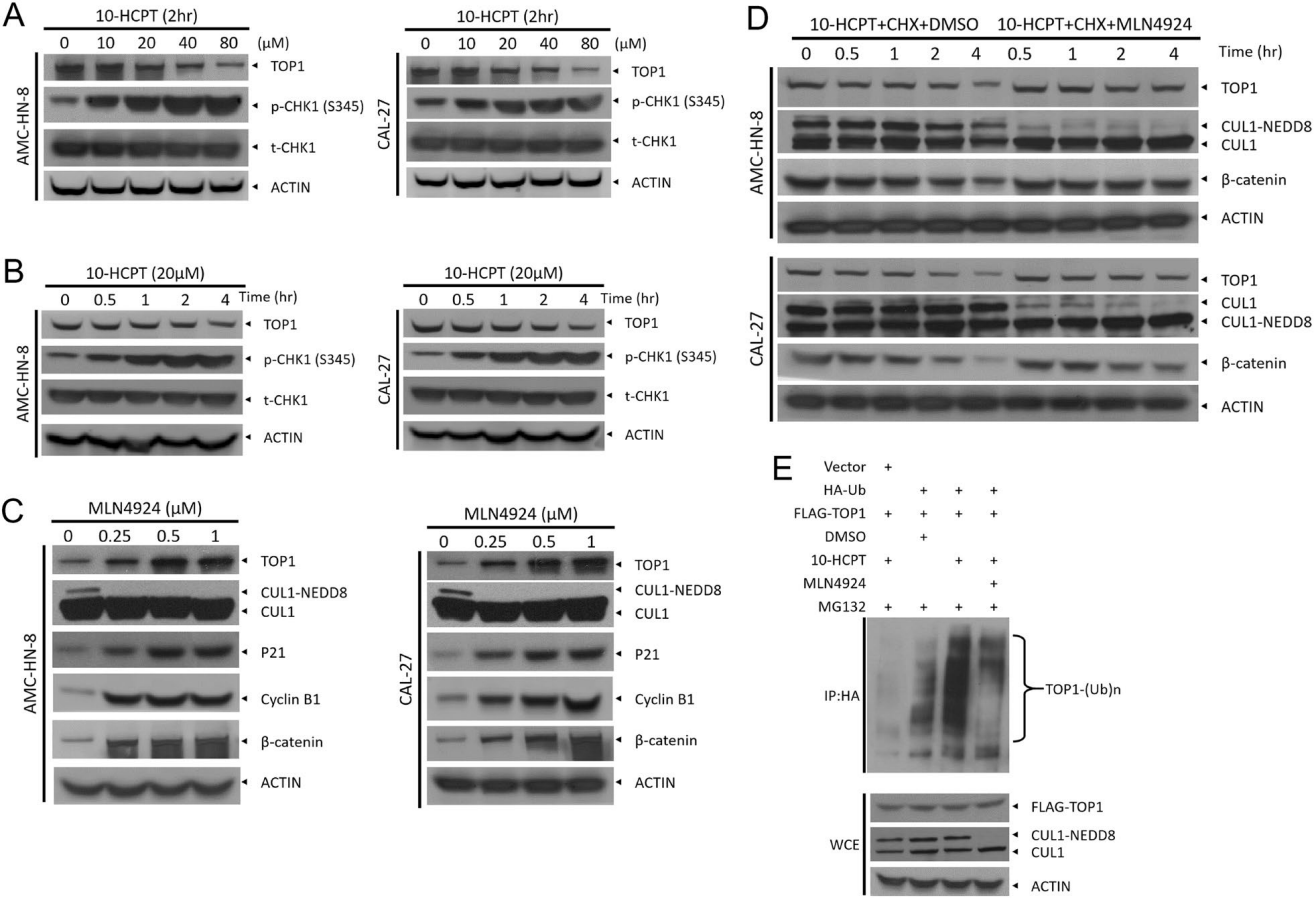
MLN4924 causes dose- and time-dependent accumulation of TOP1 and blocks TOP1 ubiquitination. **A** AMC-HN-8 and CAL-27 cells were treated with different doses of 10-HCPT (0, 10, 20, 40, and 80 µM) for 24 h, and then subjected to western blotting using antibodies against TOP1, p-CHK1 (S345), and t-CHK1. Actin was used as a loading control. **B** AMC-HN-8 and CAL-27 cells were treated with 20 µM 10-HCPT for 0, 0.5, 1, 2, and 4 h. **C** MLN4924 increased TOP1 levels in a dose-dependent manner. Cells were treated with various concentrations of MLN4924 (0, 0.25, 0.5, and 1 µM) for 24 h, and then western blotting was performed using TOP1, CUL1, p21, cyclin B1, and β-catenin antibodies. **D** MLN4924 extended the half-life of TOP1 following 10-HCPT treatment. Cells were treated with CHX (100 μg/ml) or 10-HCPT (40 μM), alone or in combination with MLN4924 (1 μM) for the indicated time periods, and then incubated with the indicated antibodies. **E** MLN4924 treatment attenuated TOP1 polyubiquitination induced by 10-HCPT treatment. HEK293 cells were transfected with the indicated plasmids for 48 h and then treated with 10-HCPT (40 μM) and MG132 (20 μM) alone or in combination with MLN4924 (1 μM) for 5 h. Immunoprecipitation with anti-HA beads was followed by IB with the indicated antibodies. WCE, whole-cell extract

HCPT, a specific inhibitor of TOP1, has shown profound anti-cancer effects with relatively low toxicity. We found that 10-HCPT treatment at various concentrations or for various durations inhibited TOP1 protein expression and caused DNA damage, as evidenced by an increase in CHK1 phosphorylation on S345 (Fig. 2A, B). Mechanistically, it is now known that the topoisomerase I–DNA covalent complex can be targeted for Cul3-regulated proteasomal degradation. We thus hypothesized that MLN4924 could be a potent sensitizer of tumor cells to 10-HCPT. First, we treated AMC-HN-8 and CAL-27 cells with various concentrations of MLN4924, which blocked neddylation of cullins and caused dose-dependent accumulation of TOP1, accompanied by increases in levels of p21, cyclin B1 and β-catenin (Fig. 2C). In addition, when cycloheximide (CHX) was used to block new protein synthesis, MLN4924 extended the half-life of TOP1 following 10-HCPT treatment (Fig. 2D). Consistently, MLN4924 treatment significantly inhibited the polyubiquitination of TOP1 induced by 10-HCPT (Fig. 2E). Collectively, these results demonstrate that MLN4924 can block the degradation of TOP1.

### MLN4924 enhances the cytotoxicity of 10-HCPT to HNSCC cells

**Fig. 3:**
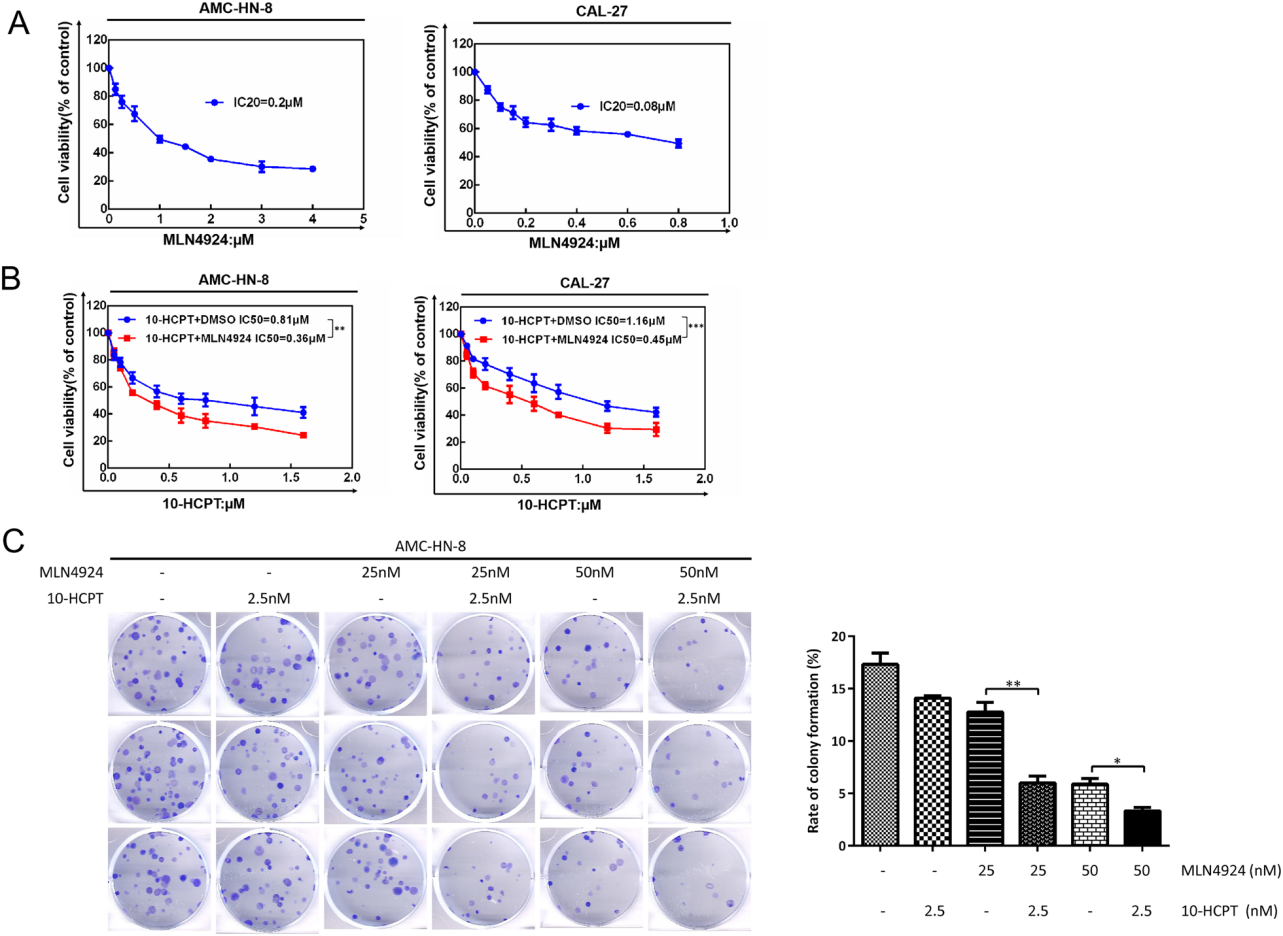
MLN4924 enhances the cytotoxicity of 10-HCPT to HNSCC cells. **A** AMC-HN-8 and CAL-27 cells were treated with various concentrations of MLN4924 for 72 h, followed by ATP-lite assay. **B** Cells were treated with various concentrations of 10-HCPT alone or in combination with an IC_20_ concentration of MLN4924, followed by ATP-lite assay. **p < 0.01, ***p < 0.001 (n = 3). **C** AMC-HN-8 cells were seeded in triplicate in 60-mm dishes at 350 cells per dish and treated with the indicated concentrations of MLN4924 (0, 25, 50 nM) alone or in combination with 10-HCPT (2.5 nM) for 7–14 days; Then, colonies were quantified. *p < 0.05, **p < 0.01 (n = 3)

To assess the ability of MLN4924 as a novel chemosensitizer to enhance 10-HCPT-mediated suppression of growth of HNSCC cells, AMC-HN-8 and CAL-27 cells were treated with various concentrations of MLN4924. According to the ATP-lite cell viability assay, the IC_20_ values for AMC-HN-8 and CAL-27 were 0.2 µM and 0.08 µM, respectively (Fig. 3A). Compared with the 10-HCPT-only group, the IC_20_ value for MLN4924 in combination with 10-HCPT showed a more dramatic decline in half-maximal inhibitory concentration values of 10-HCP from 0.81 µM to 0.36 µM and 1.16 µM to 0.45 µM, respectively (Fig. 3B). AMC-HN-8 cells were treated with MLN4924 (0, 25, and 50 nM) alone or in combination with 10-HCPT (2.5 nM) for 7 days. MLN4924 treatment potently inhibited colony formation. Moreover, the combination group (MLN4924 + 10-HCPT) showed markedly decreased clonogenic survival (Fig. 3C). These results indicate that MLN4924 can enhance the cytotoxicity of 10-HCPT and inhibit the viability of HNSCC cells.

### MLN4924 enhances the suppression of migration of HNSCC cells by 10-HCPT

**Fig. 4:**
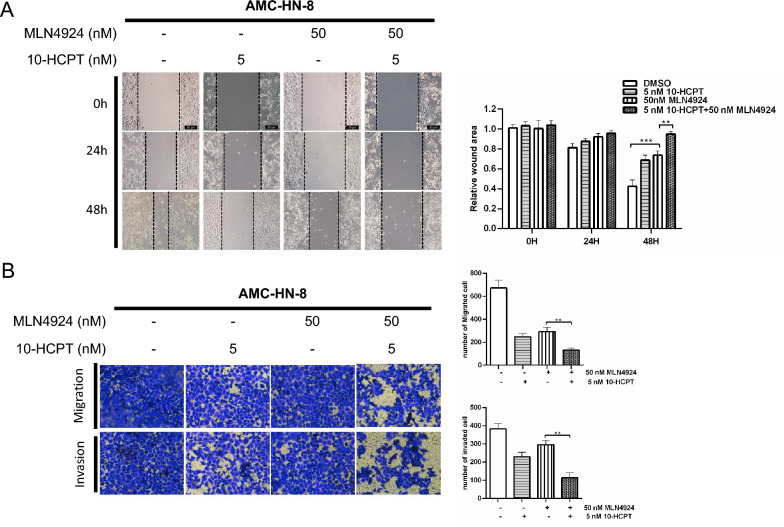
MLN4924 enhances the suppression of migration in HNSCC cells by 10-HCPT. **A**, **B** Cells were treated with MLN4924 (50 nM) and/or 10-HCPT (5 nM) for 24 h and then subjected to wound-healing analysis and transwell migration and invasion assays. ***p < 0.001, **p < 0.01 (n = 3); two-tailed unpaired Student’s *t*-test

High rates of loco-regional metastasis and recurrence account for the poor prognosis of HNSCC patients. Here, we investigated whether MLN4924 could enhance 10-HCPT-mediated suppression of migration in HNSCC. First, a wound-healing assay showed that the combination of MLN4924 and 10-HCPT was not only more effective than either drug alone but also resulted in more significant suppression of cell migration after 48 h (Fig. 4A). Importantly, results of the transwell migration and invasion assays suggested that the combination group exhibited a greater reduction in cell migration compared with the MLN4924-only or 10-HCPT-only group (Fig. 4B). Collectively, these data reveal that MLN4924 can enhance the suppression of migration induced by 10-HCPT.

### A combination of MLN4924 and 10-HCPT promotes apoptosis of HNSCC cells

**Fig. 5:**
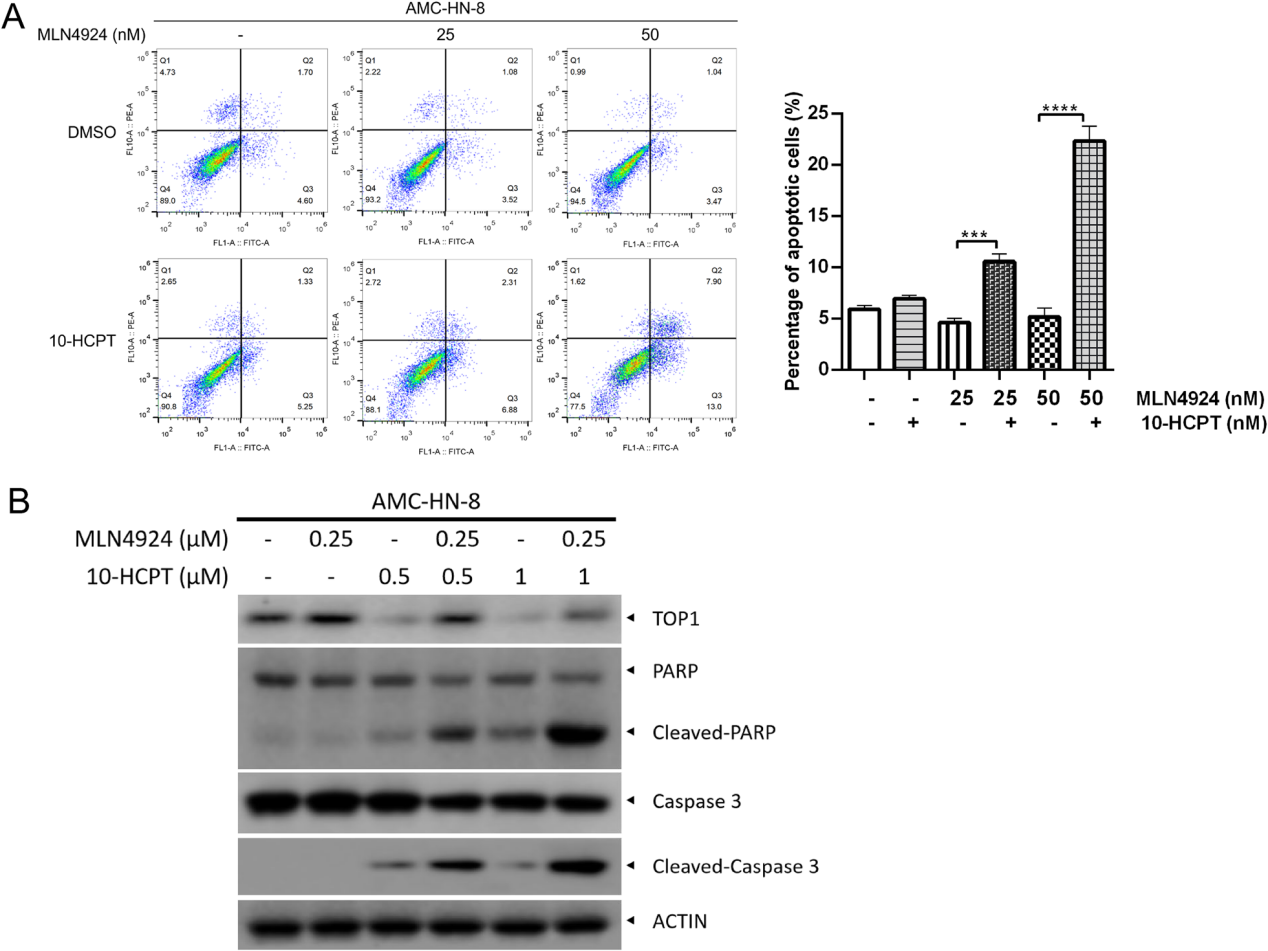
Combination of MLN4924 and 10-HCPT promotes apoptosis of HNSCC cells. **A** Cells were treated with MLN4924 (50 nM) alone or in combination with 10-HCPT (5 nM). Cell apoptosis was analyzed by flow cytometry (mean ± SEM, n = 3). **B** Cells were treated with various concentrations of MLN4924 (0, 0.25 µM) alone or in combination with 10-HCPT (0, 0.5, or 1 µM) for 24 h and then subjected to western blot analysis using the indicated antibodies

Given that 10-HCPT stabilized the topoisomerase–DNA complex, resulting in apoptotic cell death, we investigated whether MLN4924 and 10-HCPT could synergistically induce cell apoptosis. To confirm this, fluorescence-activated cell sorting analysis and western blotting were performed. As shown in Fig. 5A, the percentage of apoptotic cells, as reflected by flow cytometry of Annexin V + cells, increased sharply in the combination group compared with the single-treatment groups (Fig. 5A). Similarly, western blotting analysis showed accumulation of the cleaved forms of PARP and caspase-3 in the combination group (Fig. 5B). Taken together, these findings confirm our hypothesis that MLN4924 synergistically enhances 10-HCPT-induced apoptosis.

### MLN4924 combined with 10-HCPT had synergistic cytotoxic effects on AMC-HN-8 cells

**Fig. 6:**
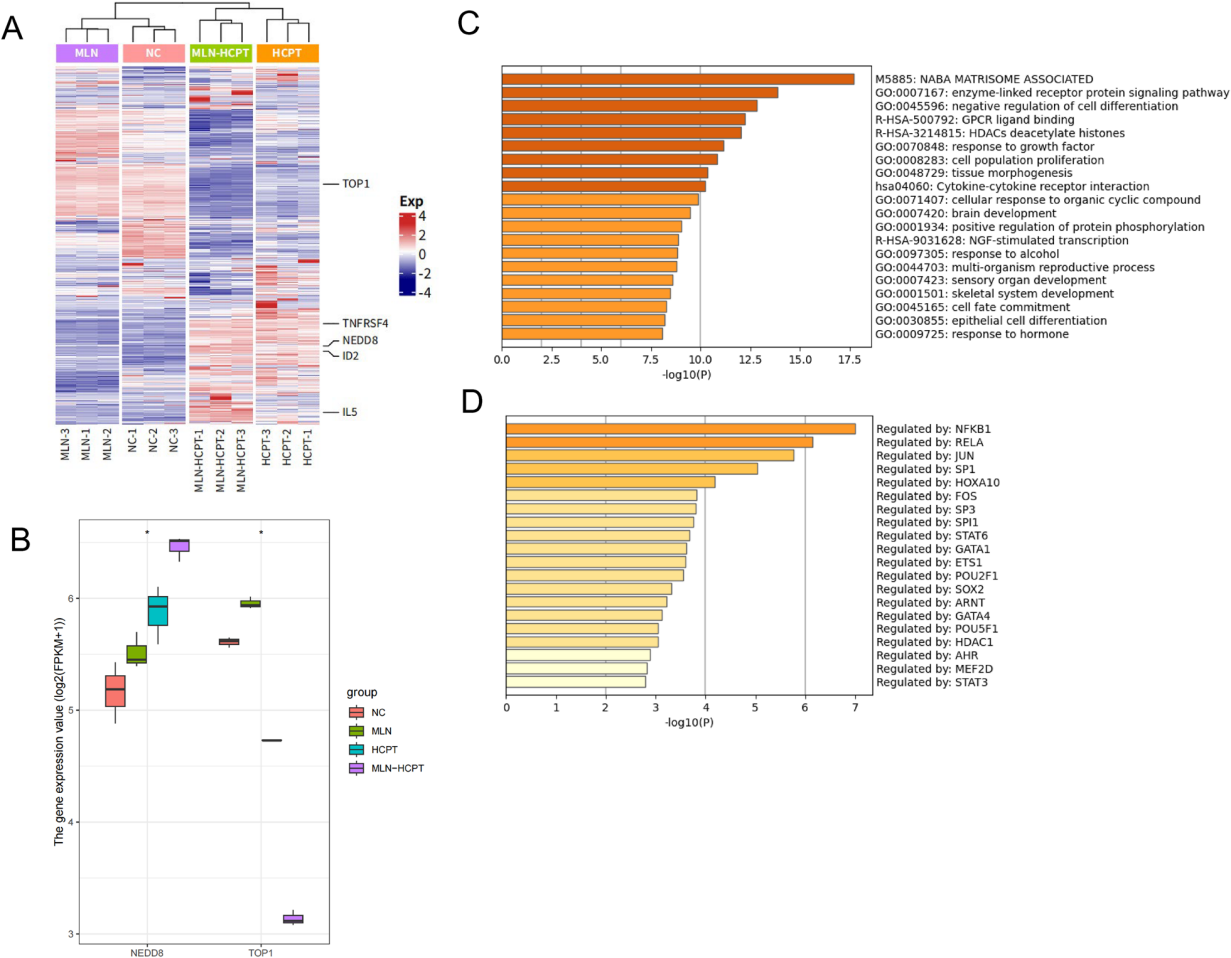
MLN4924 combined with 10-HCPT has synergistic cytotoxic effects on AMC-HN-8 cells. **A** Heat map showing gene expression of AMC-HN-8 from RNA sequencing after treatment with 10-HCPT with or without MLN4924 for 24 h. **B** Box plot showing gene expression of NEDD8 and TOP1 in different experimental groups [log2(FPKM + 1)]; *p < 0.05. **C** Bar plot of gene ontology enrichment analyses of upregulated genes in the combination group. **D** Summary of enrichment analysis in TRRUST

To further investigate the underlying mechanism of the combined treatment, we performed transcriptome sequencing on AMC-HN-8 cells. Figure 6A shows a heat map of the changes in gene expression; the group treated with 10-HCPT showed reversed gene expression relative to the MLN4924 or control group (Fig. 6A). Differential analysis revealed that TOP1 expression was significantly suppressed following 10-HCPT treatment, particularly in combination with MLN4924, indicating MLN4924 as an effective sensitizer for 10-HCPT (Fig. 6B). By contrast, NEDD8 was significantly activated. Function enrichment analyses demonstrated that the genes upregulated in the combination group were related to enzyme-linked receptor protein signaling pathways, negative regulation of cell differentiation, and GPCR ligand binding (Fig. 6C). These genes were also regulated by transcription factors including NFKB1 and RELA (Fig. 6D). By contrast, genes downregulated in the combination group were enriched in Rho GTPase cycle, Rap1 signaling pathway, etc., and regulated by transcription factors including IRF7, IRF3, IRF1, and STAT1 (Additional file 1: Fig. S1A, B). These findings suggest that the combination of MLN4924 and 10-HCPT may activate the NFKB1 pathway.

## Discussion

HNSCC represents a heterogeneous collection of malignancies of the upper aerodigestive tract, salivary glands, and thyroid [17]. Despite advances in surgery, radiotherapy, and chemotherapy, the survival of HNSCC patients has remained virtually unchanged in the past few decades [18]. Therefore, the investigation of more efficient therapeutic agents that target tumor-specific genes is attracting attention.

TOP1 has a critical role in DNA transcription and replication in both prokaryotes and eukaryotes. The elevated expression of TOP1 in various tumor cell types makes it an important drug target for anti-cancer therapy. 10-HCPT, a derivative of camptothecin, has been confirmed to have therapeutic value against various types of cancer by numerous experimental and clinical investigations [19]. It functions via topoisomerase inhibition and subsequent induction of apoptosis and autophagy [20, 21]. Advantage of 10-HCPT over camptothecin, the first-discovered TOP1 inhibitor, include its low toxicity and improved water solubility. However, two camptothecin derivatives approved by the Food and Drug Administration, topotecan and irinotecan, caused unwanted side-effects including myelosuppression. Thus, hypersensitizing cancer cells to TOP1 may be a useful therapeutic strategy. A combination of 10-HCPT with triptolide has been shown to exert enhanced anti-tumor effects by promoting apoptosis and inducing cell cycle arrest [22]. Another study found that genistein synergistically enhanced ATM/NF-κB/IKK pathway-induced apoptosis when combined with HCPT treatment [6]. In addition, studies have demonstrated that 10-HCPT can induce apoptosis via regulation of the ERK, p38, MAPK, and AKT signaling pathways [23, 24]. Encapsulation of HCPT in nanoparticles results in high stabilizing efficiency and improved anti-tumor efficacy in vivo. Furthermore, proteasome inhibitor bortezomib enhances the anti-tumor efficacy of irinotecan in mice and is currently undergoing clinical trials [25, 26]. Developing combination strategies with low toxicity that target TOP1 and NEDD8 is a new strategy.

Consistent with previous studies, we found that NEDD8 was elevated in HNSCC compared with normal tissues, possibly owing to a more active UPS system and metabolism in cancer cells. The NEDD8 level also predicted a favorable prognosis. Therefore, we chose MLN4924, a small-molecule inhibitor of NEDD8-activating enzymes that can block neddylation of all cullins and induce CRL substrate accumulation, as an adjuvant therapy for use with 10-HCPT against HNSCC. Data indicate that MLN4924 could be used to sensitize tumors to chemotherapy with drugs including carboplatin [27, 28], doxorubicin [29], and CDDP [30] in several cancers. For example, MLN4924 induces G2 cell cycle arrest and DNA damage in esophageal squamous cell carcinoma cells and sensitizes them to cisplatin [31]. Moreover, MLN4924 synergizes with carboplatin to inhibit renal medullary carcinoma cells by inhibiting DNA damage repair [32]. Likewise, treatment with MLN4924 was found to sensitize HNSCC cells to ionizing radiation (IR) and enhanced IR-induced xenografts inhibition in nude mice [33].

In the present study, we have demonstrated the following: (1) MLN4924 causes dose- and time-dependent accumulation of TOP1 and blocks TOP1 ubiquitination; (2) MLN4924 effectively enhances the suppression of cell growth, migration, and apoptosis by 10-HCPT in HNSCC cells; and (3) the combination of MLN4924 and 10-HCPT may function via the NFKB1 pathway, which would partially explain the induction of apoptosis caused by NF-κB inactivation for IkBα accumulation. Overall, our findings represent proof-of-concept evidence for future development of drug combinations involving with MLN4924 and 10-HCPT to improve cancer treatment.

### Supplementary Information

**Additional file 1: Figure S1.** (A) Bar plot of gene ontology enrichment analyses of downregulated genes in the combination group. (B) Summary of enrichment analysis in TRRUST.

## Data Availability

The datasets generated during and/or analysed during the current study are available from the corresponding author on reasonable request.
