# Supplementary material for: Neddylation inhibitor MLN4924 sensitizes head and neck squamous carcinoma cells to (S)-10-hydroxycamptothecin
Source: Eur J Med Res. 2023 Sep 9;28:326. doi: 10.1186/s40001-023-01289-y (PMC10492332; doi:10.1186/s40001-023-01289-y)
Supplement: Supplementary file 1 — Additional file 1: Figure S1. (A) Bar plot of gene ontology enrichment analyses of downregulated genes in the combination group. (B) Summary of enrichment analysis in TRRUST. [file 40001_2023_1289_MOESM1_ESM.docx]

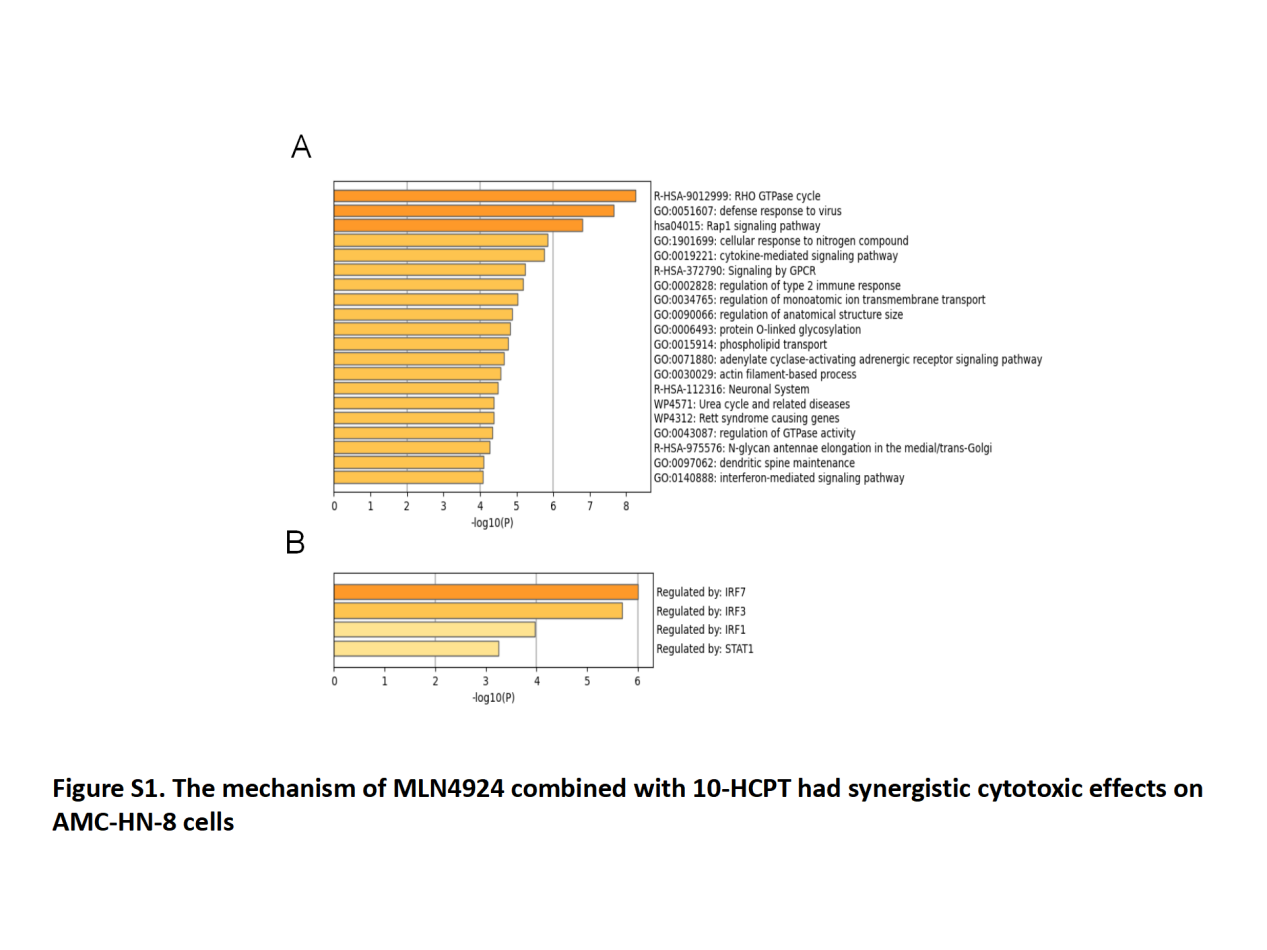


**Figure S1**

(A) Bar plot of gene ontology enrichment analyses of downregulated genes in the combination group.

(B) Summary of enrichment analysis in TRRUST.
